# A Recognition-Molecule-Free Photoelectrochemical Sensor Based on Ti_3_C_2_/TiO_2_ Heterostructure for Monitoring of Dopamine

**DOI:** 10.3390/bios13050526

**Published:** 2023-05-07

**Authors:** Zhifang Wu, Fangjie Han, Tianqi Wang, Liwei Guan, Zhishan Liang, Dongxue Han, Li Niu

**Affiliations:** 1 School of Economics and Statistics c/o Center for Advanced Analytical Science, c/o School of Chemistry and Chemical Engineering, Guangzhou Key Laboratory of Sensing Materials and Devices, Guangdong Engineering Technology Research Center for Photoelectric Sensing Materials and Devices, Guangzhou University, Guangzhou 510006, China; wzf@gzhu.edu.cn (Z.W.);; 2 Guangdong Provincial Key Laboratory of Psychoactive Substances Monitoring and Safety, Anti-Drug Tethnology Center of Guangdong Province, Guangzhou 510230, China

**Keywords:** photoelectrochemical, MXene, dopamine sensor

## Abstract

Herein, a novel, recognition-molecule-free electrode based on Ti_3_C_2_/TiO_2_ composites was synthesized using Ti_3_C_2_ as the Ti source and TiO_2_ in situ formed by oxidation on the Ti_3_C_2_ surface for the selective detection of dopamine (DA). The TiO_2_ in situ formed by oxidation on the Ti_3_C_2_ surface not only increased the catalytically active surface for DA binding but also accelerated the carrier transfer due to the coupling between TiO_2_ and Ti_3_C_2_, resulting in a better photoelectric response than pure TiO_2_. Through a series of experimental conditions optimization, the photocurrent signals obtained by the MT100 electrode were proportional to the DA concentration from 0.125 to 400 µM, with a detection limit estimated at 0.045 µM. We also monitored DA in human blood serum samples using the MT100 electrode. The results showed good recovery, demonstrating the promising use of the sensor for the analysis of DA in real samples.

## 1. Introduction

Dopamine (DA) is an important neurotransmitter that plays a key role in many physiological processes, including nerve signaling, extracerebral vasodilation, and intracerebral reward processing [1]. Brain oxidative stress caused by DA can lead to chronic fatigue syndrome, neurodegenerative diseases, and even Alzheimer’s disease, Parkinson’s disease, and Huntington’s disease [2,3]. These brain diseases not only seriously endanger human health, but also bring economic burden and social pressure to families, society, and countries. Therefore, the quantitative determination of DA is very important for early clinical diagnosis. To date, several analytical methods are available for DA determination, including fluorescence, high-performance liquid chromatography, spectrophotometry, chemiluminescence, and electrochemical and capillary electrophoresis. However, most of these techniques are expensive and involve complex operation [4,5]. Although the electrochemical detection of DA is simple in instrumentation, it is susceptible to interference by co-existing electroactive substances in the DA redox process.

Photoelectrochemical (PEC) sensing is a new and promising sensing modality that combines the advantages of both optical technology and electrochemical methods, exhibiting superior sensitivity, rapid response, low cost, and easy operation [6,7,8,9]. Meanwhile, the main process of the PEC analysis method is that the photoelectric material is in an excited state after absorbing photons under light conditions and simultaneously generates carriers and induces electron-hole separation, finally generating a photocurrent signal. Therefore, semiconductor material with an excellent optical response is the key factor affecting the PEC sensing performance.

TiO_2_ is widely used due to its excellent chemical and optical stability, nontoxicity, and low preparation cost for PEC sensors [10]. According to previous literature reports, in TiO_2_ microcrystals, the Ti atoms are natively octahedral hexacoordinated, while they are ortho-conical pentacoordinate at the surface. In addition, DA has a great affinity for sites undercoordination on the surface of TiO_2_ microcrystals. DA molecules can bind directly to TiO_2_ by diphthongal chelate bonding, restoring the Ti atoms to octahedral coordination and forming irreversible ligand–particle charge-transfer complexes [11,12]. Therefore, materials based on TiO_2_ nanostructures appear to be the most suitable as highly efficient photoactive materials to construct a recognition-molecule-free PEC sensor for DA determinations [13,14,15]. Nevertheless, the wide bandgap and rapid recombination of photogenerated electron-hole pairs in pure TiO_2_ materials inhibit its practical application as a photoelectrode material [14,16]. The most effective way to solve the above limitation is to synthesize TiO_2_ composite to form heterojunctions with other semiconductors or to perform elemental doping [17,18,19].

In recent years, two-dimensional (2D) transition metal carbides nitrides and carbonitrides (MXenes) have attracted significant international attention in various fields, such as electromagnetic shielding, supercapacitors, and sensors, due to their metal-like electrical conductivity, unique two-dimensional structures, large specific surface areas, abundant active sites, and excellent photoelectronic properties [20,21,22,23]. M_n+1_X_n_T_x_ (MXene) is usually prepared by etching the precursor M_n+1_AX_n_ (*n* = 1–3), where M represents a transition metal such as Ti, Nb, Ta, or Mo; A represents Si, Ga, or Al; X represents C or N; and Tx represents -O, -F, or -OH [24]. The composition and surface properties of MXene vary with different synthesis methods, and the most studied one is Ti_3_C_2_ produced by etching and removal of the Al layer from Ti_3_AlC_2_ [22]. Pure Ti_3_C_2_ tends to show almost no photoelectric response in PEC applications due to its metallic properties, but compounding with other semiconductors can easily form a Schottky junction, which can greatly improve electron migration efficiency and hence PEC performance [7,25]. For example, the Tang group proposed the synthesis of (001)TiO_2_/Ti_3_C_2_ heterojunctions using a hydrothermal oxidation process with NaBF_4_ to enhance the photoelectric performance [26] The TiO_2_ in situ formed by oxidation on the Ti_3_C_2_ surface not only increased the catalytically active surface for DA binding but also accelerated the carrier transfer due to the coupling between TiO_2_ and Ti_3_C_2_. However, most of the in situ oxidation methods of Ti_3_C_2_ require a long time for hydrothermal and the use of high temperatures.

In this work, we used Ti_3_C_2_/TiO_2_ composite as an electrode material to construct a recognition-molecule-free PEC sensor for DA detection. The Ti_3_C_2_/TiO_2_ composite synthesis method was performed in accordance with our previous study with minor modifications [25]. First, Ti_3_C_2_ was produced by etching and removal of the Al layer from Ti_3_AlC_2_. Then, Ti_3_C_2_/TiO_2_ heterojunctions were synthesized by a simple reflux method, and different degrees of in situ oxidation of Ti_3_C_2_ were achieved by varying the amount of hydrogen peroxide (H_2_O_2_) addition. The different oxidation levels of Ti_3_C_2_/TiO_2_ composites were analyzed and observed using SEM, TEM, XRD, and XPS. We studied distribution of TiO_2_ nanoparticles on the Ti_3_C_2_ substrate layer and explored the photoelectric activity of the composites as electrode materials. Then, we constructed an MT100-based PEC sensor for monitoring DA, which showed good chemical stability and a wide linear range of DA detection performance. Most importantly, the MT100-based PEC sensor successfully achieved the analysis of DA in real human blood serum samples.

## 2. Materials and Methods

### 2.1. Reagents and Materials

Ti_3_AlC_2_ (99.99%) was purchased from Jilin Science & Technology Co. Jinge-Wuhan provided the fluorine-doped tin oxide (FTO) glasses, which were then cut into 15 × 25 mm^2^ pieces. Dopamine (DA), ascorbic acid (AA), and uric acid (UA) were obtained from Sigma-Aldrich. Glutathione (GSH), cysteine (Cys), lysine (Lys), histidine (His), threonine (Thr), glucose (Glu), catechol (CC), resorcinol (RC), hydroquinone (HQ), 4-Bromocatechol (4-BrC), potassium ferricyanide, sodium dihydrogen phosphate, and dipotassium hydrogen phosphate were purchased from Aladdin. The human blood serum was purchased from Shanghai Sangon Biotechnology Co. All chemicals were used without any additional purification.

### 2.2. Preparation of Ti_3_C_2_/TiO_2_ Composites

The Ti_3_C_2_ MX layer was obtained by selectively etching Al with 49% HF [16,22]. First, 1.0 g Ti_3_AlC_2_ powder was soaked in 20 mL HF aqueous solution and magnetically stirred at 25 °C for 4 h. The resulting suspension was then washed several times with deionized water and centrifuged for removing excess HF until a pH value of 5–6 was obtained. The mixture was dried under a vacuum overnight at 60 °C. Next, the as-prepared Ti_3_C_2_/TiO_2_ composites were prepared by an H_2_O_2_ oxidation procedure [25]. First, 0.1 g Ti_3_C_2_ was immersed in 10 mL of deionized water, then different volumes (50 μL, 100 μL, 150 μL) of H_2_O_2_ were added, condensed, and then refluxed at 80 °C for 2 h. After cooling to room temperature, the composite was washed three times with deionized water and dried in a vacuum oven at 60 °C for 4 h.

### 2.3. Preparation of PEC Sensor

A 1.0 cm diameter hole of waterproof tape was punched on the clean FTO (2.5 cm × 1.5 cm) electrode to fix the area of the material, and then 100 μL of 2.5 mg/mL Ti_3_C_2_/TiO_2_ sample was dropped in the hole of the FTO electrode and dried at room temperature. Then, 4 mL of 0.1 M PBS buffer solution was slowly injected into the sample tube of the homemade photoelectric detection cell (Figure S1), the electrode cap was covered, the reference electrode (Ag/AgCl) and counter electrode (Pt wire) were inserted into the reserved holes, and they were connected with the electrochemical workstation. The background photocurrent signal of the sample was collected with an LED lamp alternately turned on and off from the bottom for 10 s each time. Then, the samples were tested by adding different concentrations of DA solution to the sample tube again. Each sample was tested three times, and the average value was taken.

## 3. Results and Discussion

### 3.1. Characterization of Ti_3_C_2_/TiO_2_ Composites

**Figure 1:**
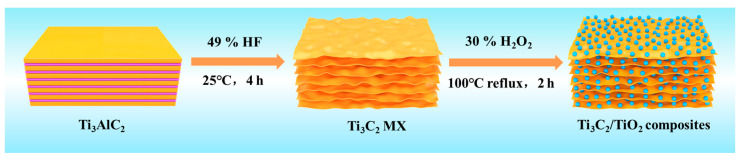
The preparation of the Ti_3_C_2_ and Ti_3_C_2_/TiO_2_ composites.

**Figure 2:**
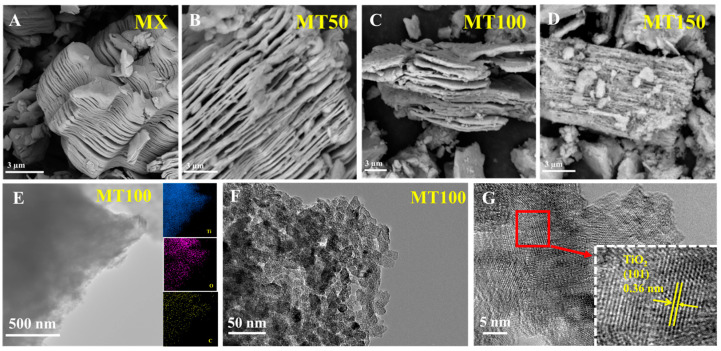
SEM image of (**A**) MX, (**B**) MT50, (**C**) MT100, (**D**) MT150. (**E**) The TEM EDS mapping, (**F**) TEM, and (**G**) HRTEM of the MT100.

Figure 1 illustrates the process of preparing the Ti_3_C_2_/TiO_2_ sample, which involved two steps: the exfoliation of the Ti_3_AlC_2_ to obtain Ti_3_C_2_, and the in situ oxidation of Ti_3_C_2_ by reflux to obtain the Ti_3_C_2_/TiO_2_ composites. The SEM and TEM were first employed to characterize the surface morphology and microstructure of the as-prepared samples. Figure 2A–D presents the SEM images of MX, MT50, MT100, and MT150 synthesized by adding different volumes of H_2_O_2_ (0 μL, 50 μL, 100 μL, 150 μL) during the in situ oxidation process, respectively. We can clearly see that the prepared Ti_3_C_2_ MX had a smooth surface and presented a cross-linked layered structure similar to an accordion. After the H_2_O_2_ oxidation treatment, the Ti_3_C_2_ MX cross-linked layers were gradually opened due to a large number of TiO_2_ nanoparticles being generated after Ti_3_C_2_ oxidation and aggregated on its surface. As the amount of H_2_O_2_ added increased during the synthesis process (50, 100, and 150 μL), the oxidation degree of Ti_3_C_2_ MX was increased and more TiO_2_ particles were generated, which can be clearly seen in the images labeled 50, 100, and 150. In addition, the TEM-EDS image of the sample (Figure 2E) shows that the elements Ti, C, and O were uniformly distributed in the selected region. High-resolution TEM (HRTEM) images exhibited lattice fringes spacing of about 0.36 nm (Figure 2G), in good agreement with the (101) plane of anatase TiO_2_ [25]. All results demonstrated the successful fabrication of Ti_3_C_2_/TiO_2_ composites.

**Figure 3:**
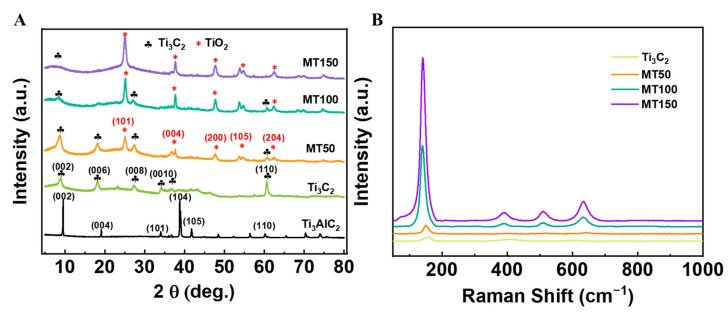
(**A**) XRD pattern of Ti_3_AlC_2_, Ti_3_C_2_, MT50, MT100, and MT150. (**B**) Raman spectra of Ti_3_C_2_, MT50, MT100, and MT150.

Meanwhile, the XRD patterns were further tested to investigate the crystalline structures of the as-prepared MX samples with various degrees of oxidation. As shown in Figure 3A, in comparison with Ti_3_AlC_2_, the diffraction line around 39° for the (104) plane of Ti_3_C_2_ did not appear, indicating the successful removal of the Al layers. In addition, a significant change in the peak position was seen when the Ti_3_C_2_ was treated with the H_2_O_2_. The bare Ti_3_C_2_ MX showed the (002) peak at 2θ = 8.8°, which is consistent with a previous report [26]. After the H_2_O_2_ was treated, the (101) and (004) peaks of anatase emerged at 25.3° and 37.8°, which matched with the standard card (PDF # 21-1272) [7]. Moreover, the (002) peak of the Ti_3_C_2_ MX phase shifted downward but remained in the sample, which corresponds to the exfoliation of Ti_3_C_2_ and indicates that more anatase TiO_2_ was formed. In Figure 3B, the Raman spectrum of Ti_3_C_2_ MX powders treated with different volumes H_2_O_2_ also showed the presence of anatase (TiO_2_). The intensity of the TiO_2_ peak around 150 cm^−1^ in the Raman spectra enhanced with the increasing amount of H_2_O_2_, which implies that the obtained products were Ti_3_C_2_/TiO_2_ composites [27,28]. At the same time, the Raman bands at around 393, 510, and 634 cm^−1^ can be ascribed to the vibration modes of nonstoichiometric titanium carbide [24,25]. In addition, it was not difficult to observe the presence of these three peaks in the MT100 and MT150 samples, and the intensity tended to increase slightly, which indicates the formation of additional TiO_2_.

**Figure 4:**
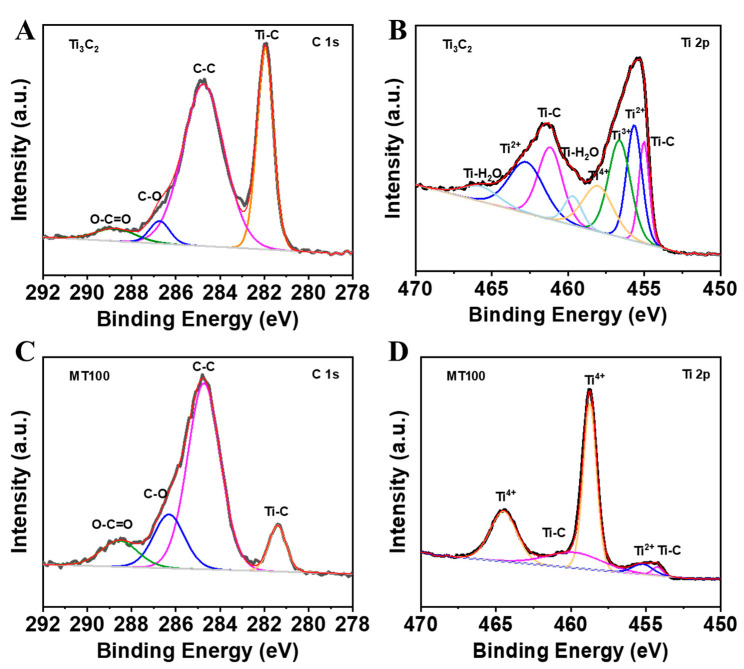
High-resolution (**A**,**C**) C 1s, and (**B**,**D**) Ti 2p XPS spectra of the as-prepared Ti_3_C_2_ and H_2_O_2_ treated MT100.

The surface chemical composition and valence state of the prepared MT100 samples were characterized by the XPS method, and the oxidation degree of titanium (Ti) was determined. Figure S2 shows the overall XPS measurement spectrum of the as-prepared Ti_3_C_2_, MT100, and MT150, and it can be seen that the main elements on the surface of the sample were titanium (Ti), fluorine (F), oxygen (O), and carbon ©, respectively. The high-resolution XPS spectra of C1s and Ti 2p of Ti_3_C_2_ and MT100 are shown in Figure 4A,C, and Figure 4B,D, respectively. The C1s’ core energy level consisted of four components with binding energies of 281.9 eV, 284.7 eV, 286.7 eV, and 288.8 eV, corresponding to Ti-C, C–C/C–H (sp3), C–O, and O–C = O [29]. It can be seen that the Ti-C peak at 281.9 eV abruptly decreased with H_2_O_2_-treated MT100. In addition, as shown in the high-resolution Ti 2p spectrum, for the as-prepared Ti_3_C_2_, 455.0 eV, 455.6 eV, 456.6 eV, 458.4 eV, 461.1 eV, and 462.7 eV were assigned to the Ti–C bond, Ti Ti^3+^, and TiO_2_, Ti–C bond, Ti^3+^ [25]. After treatment with the H_2_O_2_, two strong spin-orbit peaks at 458.7 eV and 464.5 eV can be clearly observed for the Ti 2p component mainly in the 4+ oxidation state Ti, which indicates the formation of a large number of TiO_2_ species [30]. In addition, the peaks at 455.0 eV and 459.6 eV were assigned to Ti-C 2p_3/2_ and Ti (III) 2p_3/2_, respectively. The Ti-C signal originated from the Ti atoms inside the Ti_3_C_2_ MX nanospheres (Figure 4D and Figure S2B) [31], which indicates that the Ti_3_C_2_ was not fully oxidized. This proves that the formation of TiO_2_ was at the expense of Ti_3_C_2_, and Ti_3_C_2_/TiO_2_ composites were successfully synthesized.

**Figure 5:**
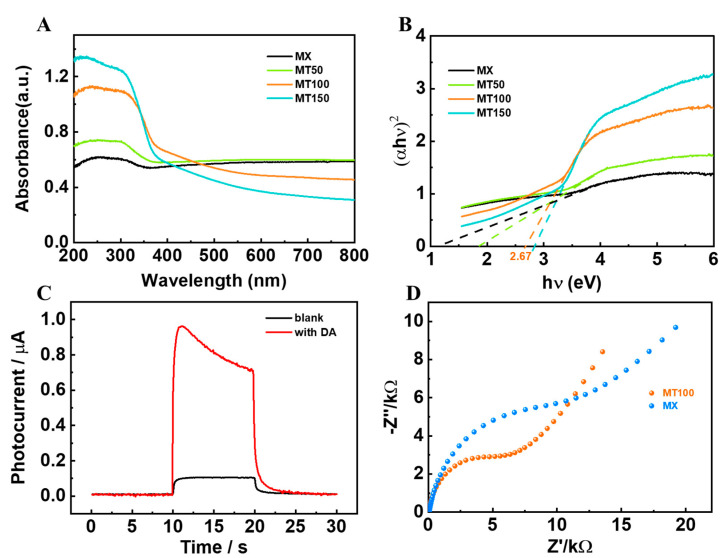
(**A**) UV−vis diffuse reflectance absorption spectra, (**B**) the corresponding K−M plot, (**C**) photocurrent responses of MT100 modified FTO electrode in the presence and absence of 50 µM DA. (**D**) EIS plots of MX and MT100 in 5 mM [Fe(CN)_6_]^3-/4-^ containing 0.1 M KCl aqueous solution.

Optical absorption information for MX, MT50, MT100, and MT150 was obtained by DRS (UV-Vis diffuse reflectance spectroscopy). In Figure 5A, the absorption intensity of pristine MX at 200–800 nm tended to be smooth and stable. However, with increasing oxidation, the absorption intensity at 200–350 nm rapidly increased, which was due to the increased content of TiO_2_ generated by oxidation, in agreement with the previously reported values. Furthermore, the K-M plot obtained by the UV-Vis DRS spectra was transformed by the Kubelka–Munk equation (Figure 5B), where the band gap (Eg) values were calculated by the intersection of the tangent line (dashed line) and the horizontal coordinate. It was observed that the band gap widened after MX oxidation, where the Eg value for MT100 was roughly estimated to be 2.67 eV.

### 3.2. Photoelectrochemical Properties

We tested the photocurrent response of the MT100−based sensor in the presence and absence of 50 µM DA, as shown in Figure 4C. In the presence of 50 µM DA, the photocurrent response was significantly enhanced about 0.749 µA, while in the absence of DA, the photocurrent response was only 0.099 µA. At the same time, we also investigated the photocurrent response of Ti_3_C_2_/TiO_2_ with different oxidation levels in the presence of 50 µM DA (Figure S3A), and the photocurrent response of a pure MX was about 0.103 µA. As the degree of oxidation increased, the photocurrent of MT50 grew to 0.201 µA. However, the photocurrent response of more oxidized MT150 (~0.511 µA) was lower compared with MT100. It was concluded that the photoelectric properties were optimal, and the MT 100 was chosen for further determining DA.

Electrochemical impedance spectroscopy (EIS) is an effective method to monitor the changes in the surface properties of the modified electrode. Figure 5D is the fitted curve of electrochemical impedance Nyquist plot curves of the modified electrodes by MX and MT100 in 5 mM [Fe(CN)_6_]^3-/4-^ solution. It can be seen that the radius of the circle of MT100 was significantly smaller than that of MX. The experimental results showed that the in situ generation of TiO_2_ on the surface of MX formed a heterojunction, which accelerated the carrier migration rate and improved the photocurrent response. Therefore, the MT100 was chosen to further the determination of DA.

In order to construct an efficient PEC sensor, some other detection conditions, such as irradiation light wavelength, bias voltage, and concentration of photodetector material, must be further optimized. As shown in Figure S3B, we selected a light source from violet to red wavelengths (365–630 nm) to test the performance of the PEC sensor in the presence of 50 µM DA, and it can be observed that the photocurrent response of MT100 to DA gradually decreased with the increasing irradiation wavelength. Moreover, the light source excitation at 365 nm showed the best photocurrent response. However, considering that visible light excitation is more favorable for the practical application of the sensor, a 420 nm visible light source was chosen. In addition, in Figure S3C, 0 V was chosen as the best voltage. Similarly, the optimal concentration of drop tinting was explored for MT100. As seen in Figure S3D, the photocurrent values gradually increased when the concentration of 100 µL MT100 was increased from 1 mg/mL to 2.5 mg/mL. However, the concentration of MT100 continued to increase, and the photocurrent value did not continue to increase, but gradually decreased. Therefore, the optimal concentration of MT100 was 2.5 mg/mL.

### 3.3. PEC Sensor for DA Detection

**Figure 6:**
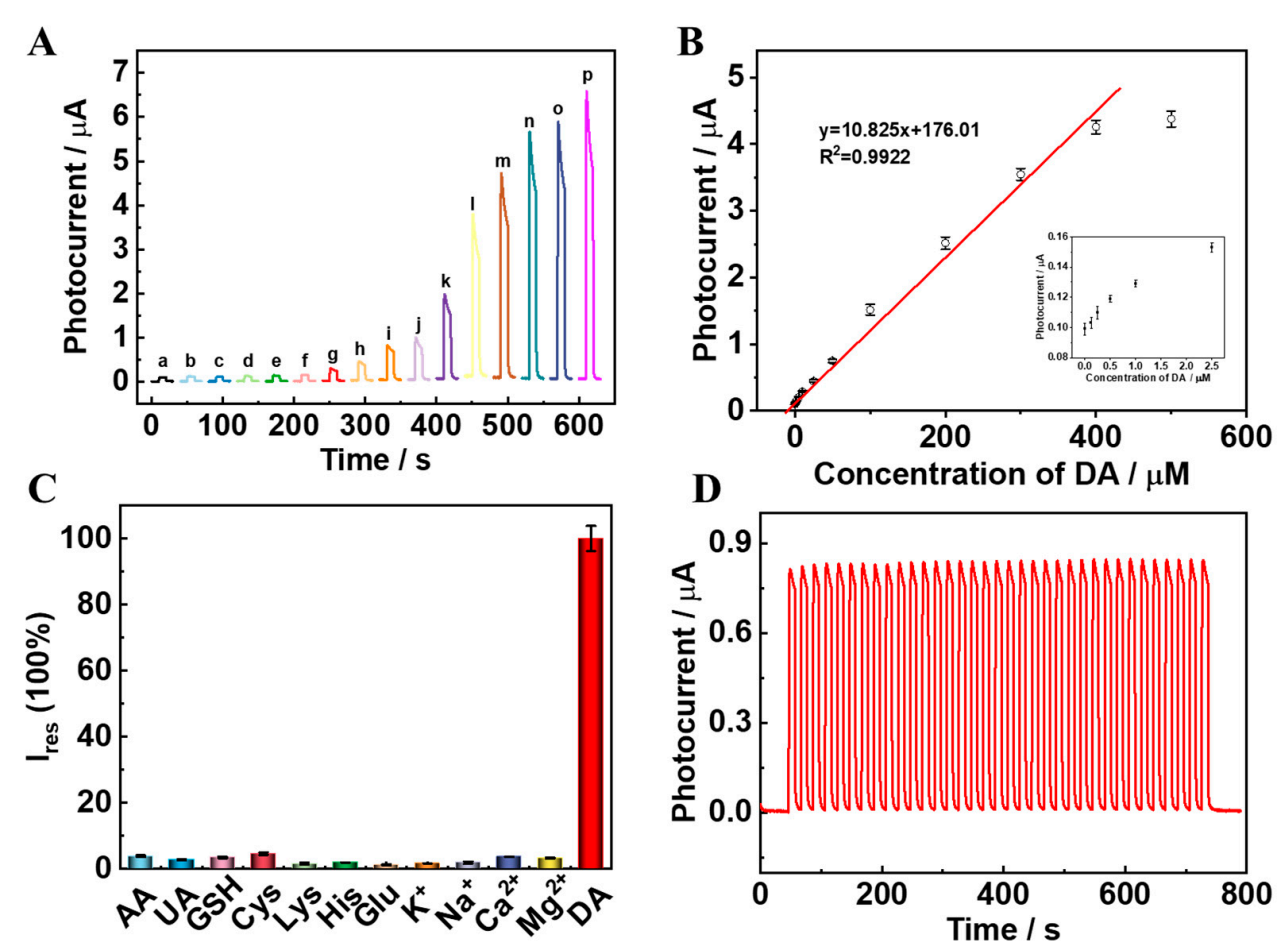
(**A**) I−t was obtained at MT100 in 0.1M PBS (pH = 7.40) with different concentrations of DA (from 0 to 1000 μM). (**B**) The plot of photocurrent values versus the DA concentrations. The insert graph is the photocurrent of the low concentration of DA. (**C**) Interface of 50 μM AA and UA, and 10 times concentration of GSH, Cys, Lys, His, Glu, K^+^, Na^+^, Ca^2+^, and Mg^2+^ with a photocurrent response of the MT100−based PEC platform in the 0.1 M PBS (pH = 7.40) containing 50 μM DA. (**D**) Stability test of the MT100−based sensor for 50 μM DA.

Under the optimized experimental conditions, a linear relationship curve was obtained by testing the photoelectric response of the sensor to different concentrations of dopamine. With the increasing concentration of DA, the photocurrent also increased accordingly. The photocurrent response curves when on/off are shown in Figure 6A. From curve a to curve p, the corresponding dopamine concentrations were 0 µM, 0.125 µM, 0.25 µM, 0.5 µM, 1 µM, 2.5 µM, 5 µM, 10 µM, 25 µM, 50 µM, 100 µM, 200 µM, 300 µM, 400 µM, 500 µM, and 1000 µM, respectively. As shown in Figure 5, the calibration curve of the dopamine sensor was fitted with the dopamine concentration as the abscissa and the photocurrent intensity as the ordinate. The results showed that the concentration of dopamine showed a good linear relationship with the photocurrent intensity from 0.125 µM to 400 µM within a certain range. The fitted linear correlation equation was y = 10.825 x + 176.01, and the correlation coefficient (R^2^) was 0.9922. The limit of detection was estimated to be 45 nM (S/N = 3) from the blank signal, the standard deviation of the blank signal, and the correlation coefficient. The detection limits were comparable to previous reports (Table S1).

### 3.4. Selectivity and Stability of the PEC Sensor

Specificity is an important indicator to assess the performance of the sensor. To investigate the specificity of the sensor, several potentially interfering substances such as ascorbic acid, uric acid, glucose, proline, lysine, threonine, and various ions were selected for testing (Figure 6C). The photocurrent response of the MT-100 based sensor in the presence of 50 µM DA was taken as 100%. It is well known that UA and AA are the most common interfering substances in DA detection because of their similar electrochemical activities [32]. The photocurrent response in the presence of 50 µM AA was only 3.9%, and that of 50 µM UA was 2.8%. In addition, there was also no obvious photocurrent response to 10-fold concentrations of other interfering substances, which did not exceed 7% compared with DA. All results verified that the excellent selectivity of our constructed MT100-based recognition-molecule-free PEC sensing platform.

Stability is quite an important factor affecting the performance and practical application of the sensor platform. After 25 on/off irradiation cycles, the photocurrent was maintained at approximately 0.751 µA (Figure 6D). There was no significant deviation in the photocurrent intensity, which means that the constructed sensor has good stability. In addition, we determined the reproducibility and repeatability of the MT100 electrode prepared by multiple synthesis in DA detection. In the reproducibility experiment (Figure S4), we can see that the photocurrent response of the five MT100s for the determination of 50 µM DA changed little, with a relative standard deviation of 2.17%. These experimental data further support the conclusion that the sensor has a better stability.

### 3.5. Real Samples Analysis

In order to further verify the feasibility of the constructed MT100 sensing platform in practical applications, the modified electrode MT100 was used to detect the content of DA in the mixed serum of healthy people. First, the photocurrent response of MT100 in PBS solution was tested. In addition, the photocurrent response of healthy human serum was tested by diluting it 50 times with PBS solution. The corresponding photocurrent in the serum sample was almost identical to that in the PBS buffer solution. Therefore, the photocurrent response signal of the serum sample was tested as a blank signal. Subsequently, DA standard solutions at different concentrations (0.5 µM, 5 µM, and 50 µM) were added by standard addition method, and each concentration was tested three times. As shown in Table 1, the recoveries of the constructed MT100 sensing platform in blood serum samples were 98.6%–102.0%, and the RSD was less than 5%, which indicates that the MT100-based sensing platform can be potentially used for DA analysis in real samples.

### 3.6. PEC Sensing Mechanism

**Scheme 1:**
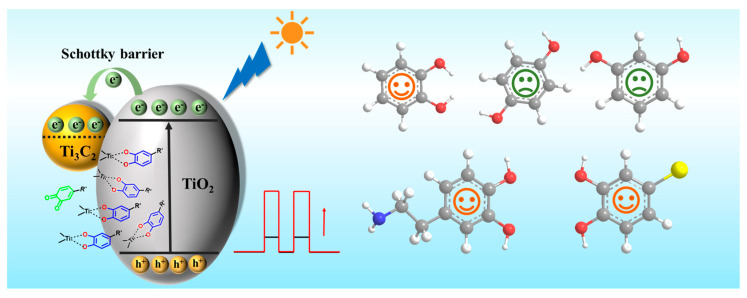
Mechanism of Ti_3_C_2_/TiO_2_−based PEC sensor for the selective detection of DA and catechol derivatives.

On the basis of the above results, the photoelectrochemical sensing mechanism for the selective detection of DA using the Ti_3_C_2_/TiO_2_ heterojunction as photoactive material was proposed in Scheme 1. When TiO_2_ was excited by visible light, a Schottky barrier was formed between the TiO_2_ nanoparticles and the Ti_3_C_2_ layer, and electrons were rapidly transfered from the conduction band (CB) of the TiO_2_ nanoparticles to the Ti_3_C_2_ layer. Due to the metallic nature of Ti_3_C_2_ MXene and the smaller size of TiO_2_ nanoparticles generated in situ on the Ti_3_C_2_ surface, the number of active sites can be increased. Moreover, the formed interfacial heterojunction can reduce the distance that holes move to Ti_3_C_2_ nanosheets during the PEC process and minimize the carrier recombination, leading to an enhanced photocurrent [33]. Recent research has shown that the o-phenyldihydroxy structure can bind directly to TiO_2_ by diphthongal chelate bonding. In order to verify the specific binding between DA and TiO_2_, we also measured the photocurrent response of some other catechol derivatives. As shown in Figure S5, the constructed MT100-based photoelectric sensor also had a good photocurrent response to CC. However, there was almost no photocurrent response to HQ and RC, indicating that the o-phenyldihydroxy structure is more likely to combine with TiO_2_ to form a complex, increasing TiO_2_ photogenerated electron-hole separation. Likewise, 4−BrC with an o-phenyldihydroxyl structure also exhibited a good photocurrent response. However, DA exhibited the highest photocurrent response because NH_2_ on DA is a very strong electron donor group. After the formation of a DA/TiO_2_ composite, the electrons on DA can transfer electrons to the conduction band of TiO_2_ through a bidentate structure, so that TiO_2_ can carry more positive charges. These positive charges are induced through an external electrical circuit, resulting in a higher photocurrent response [34]. Therefore, it was further demonstrated that the specific binding between DA and TiO_2_ occurs through a bidentate chelate bond.

## 4. Conclusions

We report a facile synthetic method to construct highly selective and sensitive photoelectrochemical sensors for the detection of DA using in situ oxidation of MXene (Ti_3_C_2_/TiO_2_) as a photoelectrode. The excellent selectivity and high sensitivity were achieved via two main processes: On the one hand, specific binding between DA and TiO_2_ occurred through a bidentate chelate bond, leading to a high selectivity for DA determination against other species. On the other hand, through the formation of interfacial heterojunctions between TiO_2_ and Ti_3_C_2_, the defect-induced carrier recombination could be minimized, resulting in improved detection sensitivity. To assess the feasibility in practical applications, the developed MT100 electrode was also used for the detection of DA in healthy human serum samples. Such a recognition-molecule-free photoelectrochemical sensor holds great promise to be applied for DA analysis in bioassays.

## Figures and Tables

**Table 1:** PEC detection of DA in the human blood serum sample.

Analyte	Added (µM)	Found (µM)	Recovery (%)	RSD (%) (*n* = 3)
Human Blood serum	0	Not detection	-	-
	0.50	0.51	102.0	4.2
	5	4.93	98.6	2.7
	50	50.6	101.2	3.5

## Data Availability

Not applicable.

## Supplementary Materials

The following supporting information can be downloaded at: https://www.mdpi.com/article/10.3390/bios13050526/s1, Figure S1: Picture of homemade photoelectric detection cell; Figure S2: XPS survey spectrum of MT100; Figure S3: Effects of (A) different degrees of oxidation of Ti_3_C_2_, (B) excitation wavelength, (C) the applied potential, and (D) the concentration of MT100 on photocurrent response of MT100-modified FTO electrode in 0.1M PBS (pH = 7.40) containing 50 μM DA; Figure S4: Photocurrent response of some other catechins derivatives on the MT100-based sensor; Table S1: Comparison of previous and current DA detection methods. References [35,36,37] are cited in the supplementary materials.
