# Supplementary material for: A Recognition-Molecule-Free Photoelectrochemical Sensor Based on Ti3C2/TiO2 Heterostructure for Monitoring of Dopamine
Source: Biosensors (Basel). 2023 May 7;13(5):526. doi: 10.3390/bios13050526 (PMC10216741; doi:10.3390/bios13050526)
Supplement: Supplementary file 1 [file biosensors-13-00526-s001.zip › biosensors-2339596-supplementary.pdf]

A recognition-molecule-free photoelectrochemical sensor based on Ti_3C_2/TiO_2 heterostructure for monitoring of dopamine

Zhifang Wu [1], Fangjie Han [1] Tianqi Wang [1], Liwei Guan [1], Zhishan Liang [1], Dongxue Han[*1, 2], and Li Niu [1]

1 School of Economics and Statistics c/o Center for Advanced Analytical Science, School of Chemistry and Chemical Engineering, Guangzhou Key Laboratory of Sensing Materials & Devices, Guangzhou University, Guangzhou 510006, P. R. China.

2. Guangdong Provincial Key Laboratory of Psychoactive Substances Monitoring and Safety， Anti-Drug Tethnology Center of Guangdong Province, Guangzhou 510230, PR China.

* Correspondence: dxhan@gzhu.edu.cn

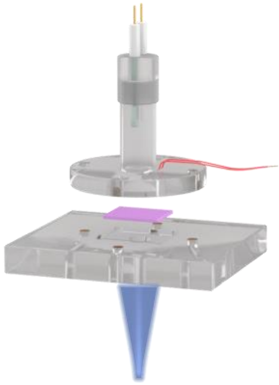

Figure S1 Picture of homemade photoelectric detection cell.

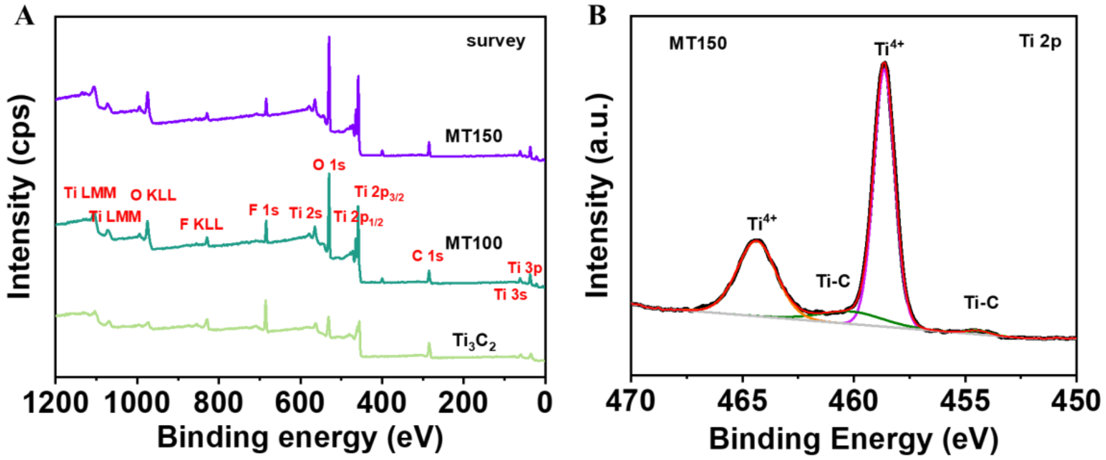

Figure S2 (A) XPS survey spectrum of Ti_3C_2, MT100, and MT150. (B) High-resolution Ti 2p XPS spectra of the MT150.

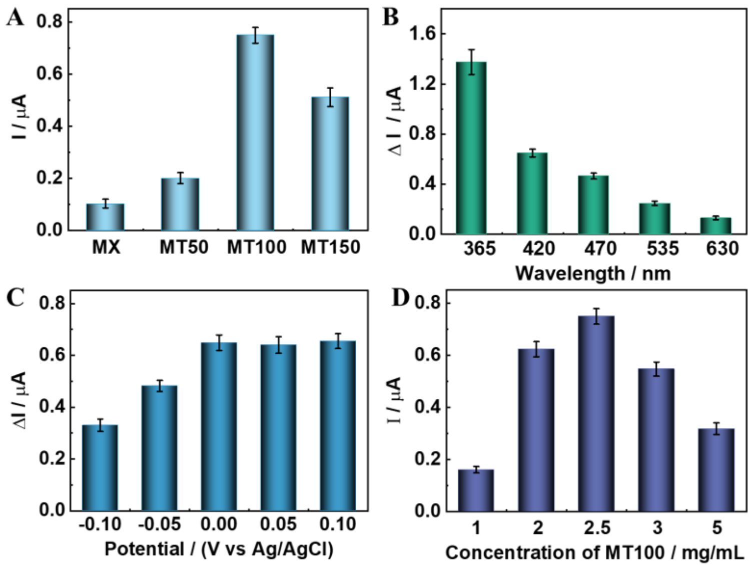

Figure S3. Effects of (A) different degrees of oxidation of Ti_3C_2, (B) excitation wavelength, (C) the applied potential and (D) the concentration of MT100 on photocurrent response of MT100-modified FTO electrode in 0.1M PBS (pH=7.40) containing 50 μM DA.

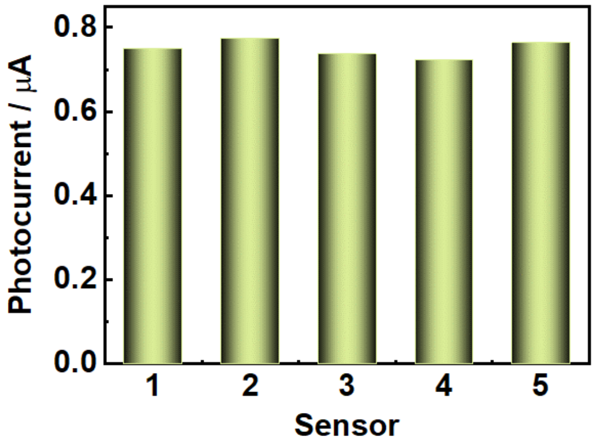

Figure S4 Repeatability of five MT100 photoelectrodes for detection of 50 μM DA.

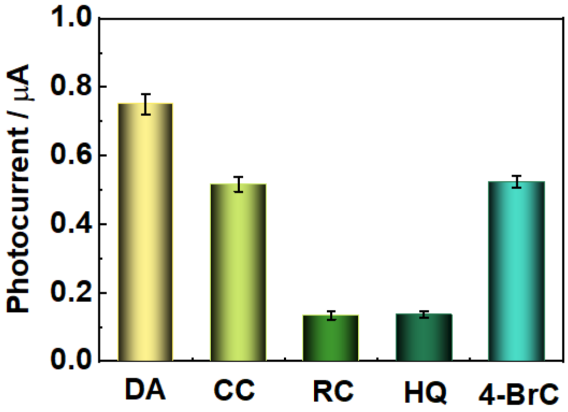

Figure S5. Photocurrent response of some other catechins derivatives on the MT100-based sensor.

Table S1 Comparison of previous and current DA detection methods

Material	Method	Linear range (μM)	Limit of detection (μM)	Refs
$BiVO_4$/FeOOH	PEC	0.2–40 and 40–1400	0.09	1
GDY@TiO_2	PEC	0.5-500	0.136	2
Cu_2O@CdTe QDs	PEC	0.001–0.100	0.0003	3
DE-TiO_2 NTPCs	PEC	0.01–2	0.002	4
WS_2/TiO_2	PEC	0.99−48.78 72.29−333.33	0.32	5
TiO_2 nanopore array	PEC	200–1500	20	6
Ti_3C_2@(001)TiO_2	PEC	1-1000	0.52	7
ZnPc-P8BT-Pdots	PEC	0.0025-125	0.00169	8
Ti_3C_2/TiO_2	PEC	0.125-400	0.045	This work

References

1. Wang, Y.; Wang, D.; Dong, S.; Qiao, J.; Zeng, Z.; Shao, S., A visible-light-driven photoelectrochemical sensing platform based on the $BiVO_4$/FeOOH photoanode for dopamine detection. *Electrochim. Acta* **2022,** *414*, 140207.

2. Xie, Y. Z.; Wang, Y.; Ma, Y.; Ye, J. S., Photoelectrochemical sensor based on carboxylated graphdiyne co-sensitized TiO_2 for sensitive detection of dopamine. *Materials Today Chemistry* **2022,** *26*, 101143.

3. Wang, C.; Chen, J.; Zhang, L.; Yang, Y.; Huang, M.; Chen, C.; Li, C.; Xie, Y.; Zhao, P.; Fei, J., An ultra-sensitive dopamine photoelectrochemical sensing platform based on two-dimensional Zn carbon nanosheets, hollow Cu_2O and CdTe QDs composite films. *Carbon* **2022,** *198*, 101-109.

4. Wu, W.; Zhang, Z., Defect-engineered TiO_2 nanotube photonic crystals for the fabrication of near-infrared photoelectrochemical sensor. *J. Mater. Chem. B* **2017,** *5*, 4883-4889.

5. Ma, W.; Wang, L.; Zhang, N.; Han, D.; Dong, X.; Niu, L., Biomolecule-free, selective detection of o-diphenol and its derivatives with WS_2/TiO_2-based photoelectrochemical platform. *Anal. Chem.* **2015,** *87*, 4844-4850.

6. Tavella, F.; Ampelli, C.; Leonardi, S. G.; Neri, G., Photo-electrochemical sensing of dopamine by a novel porous TiO_2 array-modified screen-printed Ti electrode. *Sensors* **2018,** *18*, 3566.

7. Chen, Y.; Li, X.; Cai, G.; Li, M.; Tang, D., In situ formation of (001)TiO_2/Ti_3C_2 heterojunctions for enhanced photoelectrochemical detection of dopamine. *Electrochem. Commun.* **2021,** *125*, 106987.

8. Peng, J.; Li, X.; Liu, Y.; Zhuge, W.; Zhang, C.; Huang, Y.,

Photoelectrochemical sensor based on zinc phthalocyanine semiconducting polymer dots for ultrasensitive detection of dopamine. *Sensors and Actuators B: Chemical* **2022,** *360*.
